# KLF4: a multifunctional nexus connecting tumor progression and immune regulation

**DOI:** 10.3389/fimmu.2025.1514780

**Published:** 2025-02-06

**Authors:** Yunjie Ju, Wen Xiao, Bryan James Mathis, Ying Shi

**Affiliations:** ^1^ Department of Urology, Union Hospital, Tongji Medical College, Huazhong University of Science and Technology, Wuhan, China; ^2^ Clinical Research Manuscript Elevation Service, University of Tsukuba Institute of Medicine, Tsukuba, Japan

**Keywords:** KLF4, tumor microenvironment (TME), immune regulation, oncogenesis, tumor immunology

## Abstract

Krüppel-like factors (KLFs) regulate various biological processes such as cell proliferation, migration, invasion, and differentiation as gene transcription factors. Signaling pathways which mediated by KLF4 and KLF4 have a sophisticated role in tumors due to multiple factors, including the types or stage of tumors. KLF4 plays a promoter role in tumorigenesis and development, or tumor suppressor as a context-dependent anti- and pro-inflammatory factor. KLF4 over-expression increases CD8+T cell differentiation and enhances the antitumor immunity. This review aims to provide information about the relationship of KLF4 in immunity with tumors and to guide the future study.

## 1 Introduction

Tumor-immune interactions are a critical driver of tumorigenesis and progression while also offering targets for preventive strategies or novel therapies. The tumor microenvironment (TME) and its unique immune milieu plays a dual role in both suppressing tumor growth and promoting tumor progression as multi-facted regulation is crucial for tumor immune evasion, TME formation, and the modulation of anti-tumor immune responses. However, the molecular networks connecting immunity and tumor progression are dense and interconnected, highlighting the necessity of identifying key “bridge” molecules that link immunity and tumor biology for the development of novel therapeutic strategies.

Interestingly, certain genes, such as TGF-β (1), Notch (2), Runx (3), E2f (4), CDKN1A (5), and Krüppel-like factor 4 (KLF4) (6), exhibit dual functionality in tumors. A single gene may serve as both a tumor suppressor and an oncogene in specific cancers; this paradox underscores the intricacy of tumor biology. Among dual-function transcription factors, KLF4, a prominent member of the Krüppel-like factor family, demonstrates this context-dependent dual role in tumor biology and immune system regulation. How exactly does KLF4 serve as a critical “bridge” connecting immunity and tumor biology by simultaneously regulating the tumor microenvironment and immune responses? These questions warrant deeper exploration to uncover KLF4’s potential as a pivotal molecular target in cancer immunotherapy.

KLF4 is a zinc finger-containing transcription factor belonging to the Specificity Protein (SP)/KLF family (7). The gene encoding KLF4 (*Klf4*) was first isolated by Shields et al. in 1996 (8). As a transcription factor, KLF4 plays diverse roles in the development and progression of multiple diseases across varying tissues and organs, as well as in multiple biological processes. In the context of cancer, KLF4 and its interacting molecular pathways exhibit complex, multifaceted roles depending on the type or stage of the tumor.

KLF4 is frequently linked to cancer stem cells (CSCs), which possess the ability to self-renew and differentiate into multiple tumor cell types. As CSCs are well-known contributors to cancer recurrence, metastasis, and resistance to chemotherapy tolerance, the central role of KLF4 in CSC regulation underscores its importance in tumor progression and therapy resistance (9, 10).

Despite significant advances in KLF4 regulatory mechanisms in tumorigenesis, research on KLF4’s role within the immune system and its relationship to cancer remains limited. This review aims to explore the biological and molecular mechanisms of KLF4 within the immune system, with a particular focus on its interactions with tumor immunity and its dual role as a switchable oncogene/suppressor. Additionally, this review will outline potential future research directions, emphasizing KLF4’s function in both normal immune cells and tumor immunity.

## 2 Structure and function of KLF4

**Figure 1:**
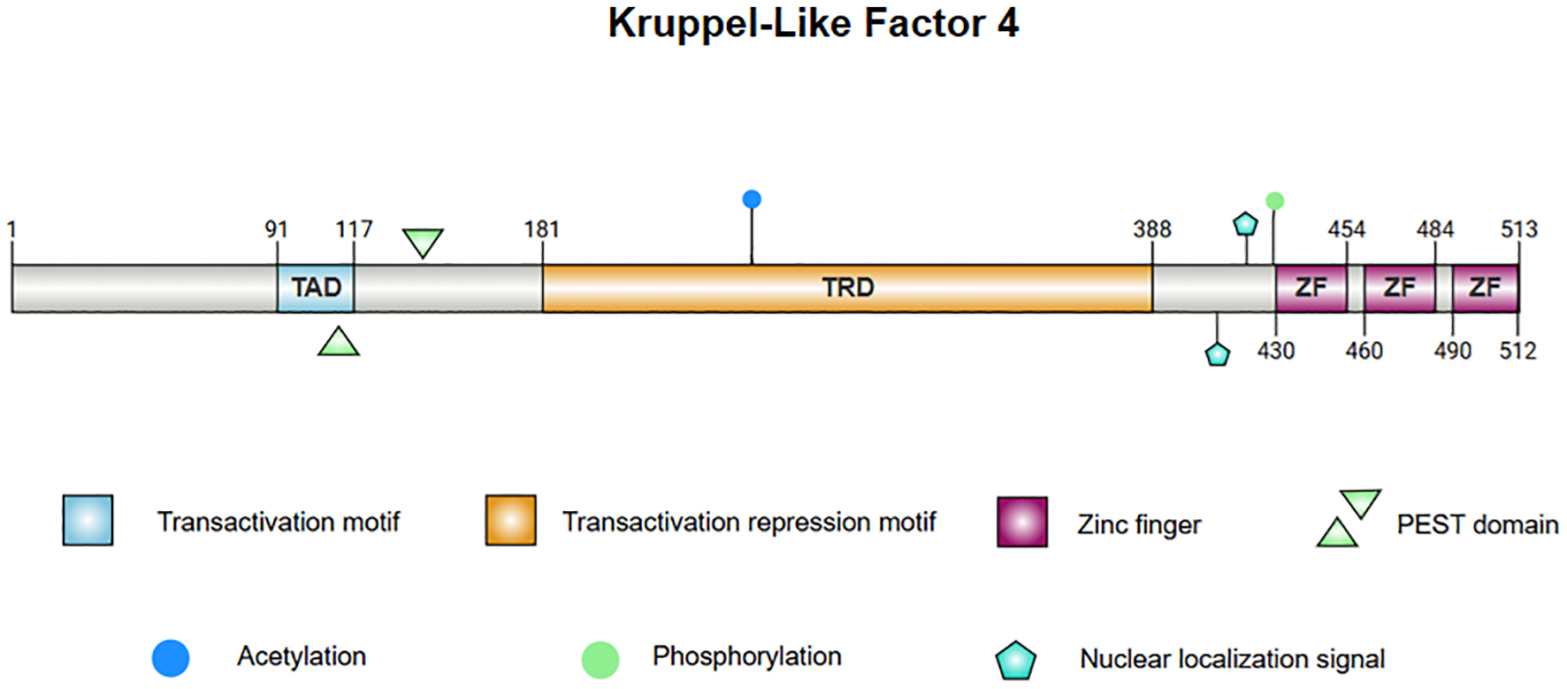
The protein structure of KLF4. 1. Linear diagram of the KLF4 protein, visualizing the protein sequence and functional regions. 2. The central image depicts the superimposed structure of KLF4 predicted by AlphaFold and the X-ray diffraction-derived protein model, demonstrating consistency in regions with high confidence. The left panel presents the AlphaFold-predicted structure of KLF4, with the Predicted Aligned Error (PAE) set to 300×300. The right panel illustrates the partial structure of KLF4 obtained from the PDB database via X-ray diffraction. Note: Internal confidence score (commonly referred to as pLDDT, Predicted Local Distance Difference Test): pLDDT > 90: Dark blue; 70 < pLDDT ≤ 90: Light blue; 50 < pLDDT ≤ 70: Yellow; pLDDT ≤ 50: Orange or red.

The structure of KLF4 is comprised of multiple functional domains that confer specific transcriptional regulatory functions. Protein databases list the canonical sequence of KLF4_HUMAN at 513 amino acids but, in contrast, most experimental functional studies utilize KLF4_MOUSE, whose canonical sequence has a standard length of 483 amino acids. With the rapid development of AI technology, particularly tools such as AlphaFold (11, 12), the protein structure of KLF4 has been predicted with high accuracy, laying the foundation for further investigation into the mechanisms of KLF4 in disease. The structure of KLF4 is shown in **Figure 1**. The *Klf4* gene is primarily composed of five exons and four introns, encoding the KLF4 protein, which has three highly conserved C2H2-type zinc finger motifs at its carboxyl terminus. These zinc finger motifs are the defining characteristic of KLF4, enabling it to bind specifically to GC-rich regions in DNA, such as CACCC boxes, thereby regulating the transcription of downstream target genes (13). The normal function of KLF4 depends on the integrity of zinc finger domain. Crystallographic studies have revealed that the first zinc finger motif inhibits KLF4’s ability to promote self-renewal and block differentiation while the two C-terminal zinc finger motifs are essential for DNA binding specificity, facilitating the terminal differentiation of macrophages. Disruption of KLF4’s DNA-binding function, as indicated by crystallographic data, may impair macrophage differentiation and has been implicated in hematologic malignancies, such as monocytic leukemia (14).

KLF4’s N-terminus contains both transcriptional activation and repression domains. A proline/serine-rich transactivation domain is located between amino acid residues 91 and 117, which promotes the expression of downstream genes. Adjacent to this is a repressive domain between residues 181 and 388, which collectively determine KLF4’s specificity in regulating transcriptional activity (15–17). KLF4 also contains two nuclear localization signals (NLS), one located near and the other upstream of the zinc finger motifs, which facilitate KLF4’s entry into the nucleus for transcriptional regulation (15). Additionally, KLF4 harbors a proline (P), glutamic acid (E), serine (S), and threonine (T) (PEST) region which is involved in protein degradation and regulates its own stability (18). In lymphomas and leukemias, mutations in this region may inhibit ubiquitination and degradation of KLF4 in tumors, contributing to malignant progression. Mutations in the PEST region may also be linked to early-stage pancreatic ductal adenocarcinoma (PDA) (19).

KLF4 contains several functional mutation sites, with certain key residues responsible for phosphorylation and acetylation modifications (8, 20, 21). These modifications affect KLF4’s stability and activity, and mutations at these sites alter its function in specific cancer contexts. For instance, the A472D mutation in KLF4 may play a critical role in the development of resistance to the anti-EGFR monoclonal antibody cetuximab in patients with metastatic colorectal cancer (mCRC) (22). Overall, this complex structure enables KLF4 to promote both stem cell differentiation and the maintenance of cellular homeostasis, while also playing a dual role in tumorigenesis.

## 3 The fundamental roles of KLF4 in the immune system

**Figure 2:**
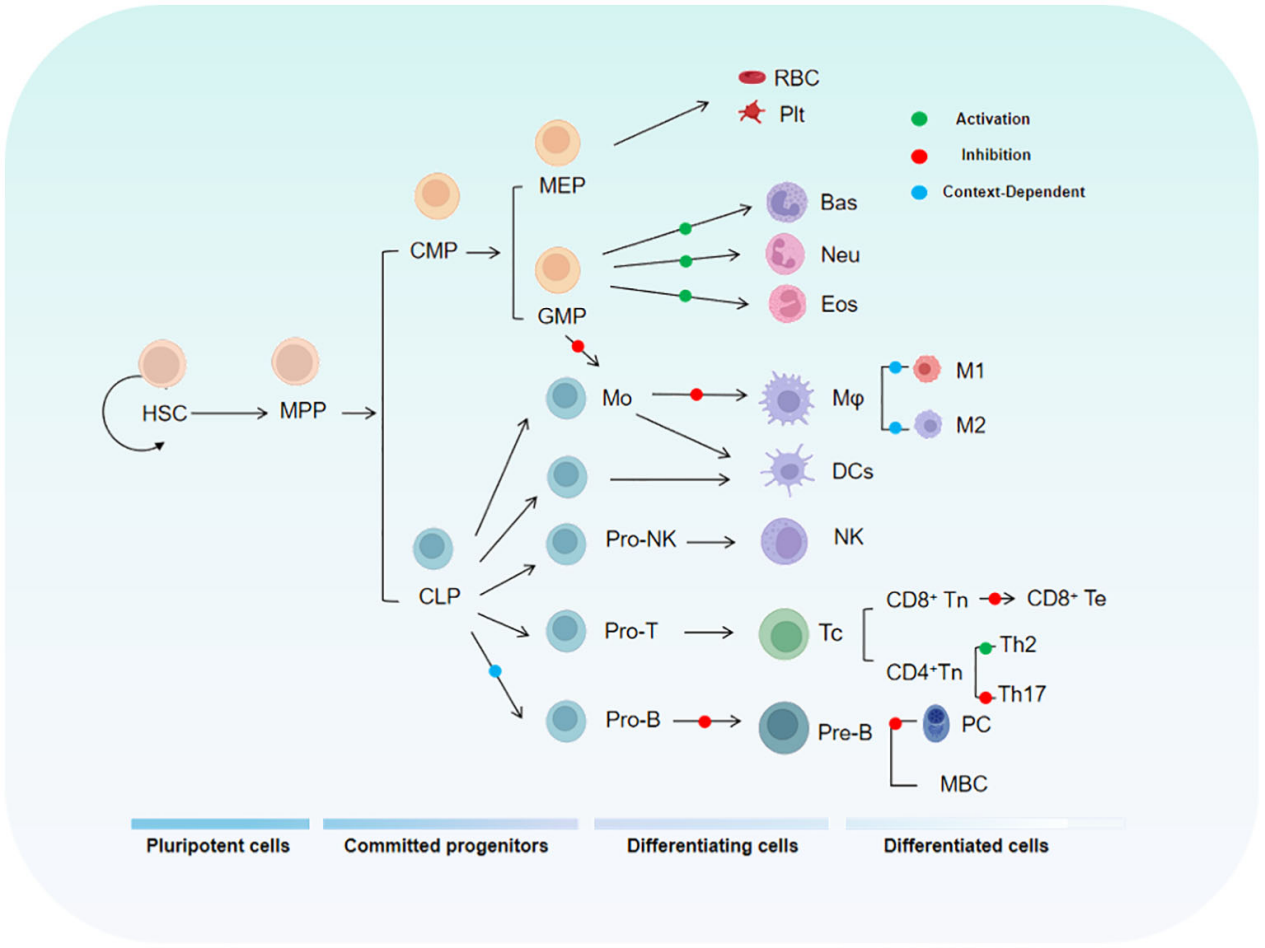
The diagram illustrates the relationship between KLF4 and the hematopoietic hierarchy. HSC, Hematopoietic Stem Cell; MPP, Multipotent Progenitor; CMP, Common Myeloid Progenitor; MEP, Megakaryocyte-Erythroid Progenitor; GMP, Granulocyte-Monocyte Progenitor.

To fully understand the role of KLF4 in tumor immunity, it is essential to first examine its functions in the normal immune system. As a critical transcription factor, KLF4 is involved in the differentiation, activation, and migration of immune cells, while also playing a key role in maintaining immune homeostasis. The relationship between KLF4 and the maturation and differentiation of immune cells during hematopoiesis is shown in **Figure 2**.

### 3.1 KLF4 and T lymphocytes

During T cell development, multiple transcription factors regulate the expression of specific genes in series and stages, including hematopoiesis, thymic differentiation, and peripheral T cell specialization. KLF4 has been implicated in the differentiation, activation, quiescence, and homing of various T cell subpopulations (23). KLF4 is highly expressed in thymocytes and mature T cells but is rapidly downregulated following T cell activation (24) as this regulatory mechanism maintains a swift immune response while preventing autoimmunity. Studies have shown that specific deletion of the *Klf4* gene in CD4+ T cells in the mouse thymus significantly inhibits the differentiation of Th17 cells and the expression of IL-17, demonstrating that KLF4 directly regulates Th17 differentiation and function via potential direct binding to the *Il17a* promoter (24). *In vitro* experiments by Yamada, Mamonkin, and colleagues showed that, under normal conditions, KLF4 suppressed the proliferation of naïve CD8+ T cells, potentially through the regulation of p21 expression in T cells (25). However, subsequent *in vivo* experiments revealed that the proliferation, maturation, and distribution of naïve CD8+ T cells were not significantly affected by *Klf4* deletion. Conversely, under infection conditions, *Klf4* deletion significantly increased the proliferation of naïve CD8+ T cells and the formation of memory T cells, indicating that the regulation of CD8+ naïve and memory T cell proliferation and differentiation by KLF4 varies by circumstance and that molecular sensors exist to switch it for such purposes (24, 26). KLF4’s Lys-48-linked ubiquitination is mediated by the E3 ubiquitin ligase Mule, which is crucial for T cell entry into S phase and proliferation. When Mule is absent, KLF4 accumulates, leading to E2F2 upregulation and activation of cell cycle inhibitors p21 and p27, thereby inhibiting T cell proliferation. Additionally, KLF4 expression is reduced in CD8+ T cells lacking KLF4, a condition that promotes excessive proliferation of CD8+ T cells (27). Tussiwand and colleagues demonstrated that KLF4 is an essential factor for inducing Th2 cell responses in classical dendritic cells (cDCs) expressing IRF4 (28).

### 3.2 KLF4 and monocytes/macrophages

Monocytes and macrophages are central for both innate and adaptive immunity and are closely associated with the pathogenesis of acute and chronic inflammatory diseases, as well as cancer (29). Recent studies have shown that KLF4 is a critical regulator of monocyte commitment, differentiation, and macrophage activation (30–32). KLF4 is highly expressed in monocytes and *in vivo* experiments have demonstrated that monocytic differentiation in KLF4-deficient wild-type mice is significantly impaired, with reductions in bone marrow monocytes, as well as decreases in both resident monocytes in the spleen and circulating monocytes. This may be related to the ability of intact KLF4 to transactivate the monocyte-specific *CD14* promoter by binding to KLF common sites, thereby promoting monocyte differentiation. Reports indicate that KLF4 is expressed in a monocyte- and stage-specific manner during myelopoiesis. Forced expression of KLF4 out of sequence can induce a mature monocyte phenotype in promyelocytic HL-60 cells, characterized by significant upregulation of myeloid markers CD11b and CD14 and morphological changes in monocytes. In macrophages, Noti and colleagues found that KLF4 regulates the differentiation marker CD11d (33). KLF4 in macrophages is induced by pro-inflammatory stimuli such as IFN-γ, LPS, and TNF-α, while it is suppressed by the anti-inflammatory growth factor TGF-β1. Knockdown of KLF4 reduces the induction of inducible nitric oxide synthase (iNOS) by IFN-γ and/or LPS, while enhancing the response to TGF-β1 and Smad3 signaling. Mechanistically, KLF4 likely activates the *iNOS* promoter by binding to KLF4-binding sites and interacting with NF-κB family member p65. Additionally, KLF4 inhibits TGF-β1- and Smad3-mediated induction of plasminogen activator inhibitor-1 by competing with Smad3 for binding to the co-activator p300/CBP. In another study, acetylation of KLF4 at lysine residues 225 and 229, mediated by p300/CBP, was found to inhibit KLF4’s ability to activate downstream targets, suggesting that this specific acetylation modification limits KLF4’s transcriptional activation capacity (34). In the RAW264.7 macrophage cell line, KLF4 has been shown to positively regulate the expression of HMGB1, a late-stage inflammatory mediator. The *HMGB1* promoter contains two potential KLF4-binding sites, and transfection of KLF4 expression plasmids or KLF4 antisense oligonucleotides into RAW264.7 macrophages increased the expression of HMGB1 in both the nucleus and cytoplasm, while KLF4 deficiency led to reduced HMGB1 expression (35). Finally, research by Alder et al. showed that in KLF4-deficient chimeric mice, circulating inflammatory monocytes (CD115^+^Gr1^+^) were almost undetectable (35). CD115^+^Gr1^+^ monocytes are capable of differentiating into macrophages and migrating to sites of inflammation, suggesting that the loss of KLF4 affects the generation and migration of pro-inflammatory monocytes. This highlights the critical role of KLF4 in monocyte development and mobilization.

### 3.3 KLF4 and B lymphocytes

Research indicates that KLF4 plays an important role in B cell development and proliferation of induced B cells as t expression level of Klf4 is low in pro-B cells but progressively increases in pre-B cells (36). KLF4 induces B cell proliferation by regulating cyclin D2 and, in Klf4-deficient mice, the number of pre-B cells in the bone marrow and mature B cells in the spleen is reduced (36). In the absence of Klf4, B cell DNA synthesis decreases while G1 phase cell numbers increase, suggesting that Klf4 deficiency impedes cellular proliferation. Other studies have shown that, in B cell-specific KLF4-deficient mice, the number of pre-B cells in the bone marrow and mature B cells in the spleen is moderately reduced, while bone marrow numbers of pro-B cells remain constant (37). These differing results highlight the functional variability of KLF4 in different environments, emphasizing its multifunctionality. *In vitro* experiments by Schoenhals and colleagues demonstrated that KLF4 can induce memory B cells to finally differentiate into long-lived quiescent plasma cells. Moreover, overexpression of Klf4 can promote the differentiation of plasma blasts into early plasma cells and long-lived plasma cells by inhibiting apoptosis and upregulating pathways involved in plasma cell differentiation (38).

### 3.4 KLF4 and granulocytes

Neutrophils are the most abundant circulatory leukocytes and are key to both innate and adaptive immune responses (39, 40). Granulocytes, such as neutrophils, and macrophages originate from a common myeloid progenitor (CMP) and granulocyte/macrophage progenitors in the bone marrow (41, 42), suggesting that KLF4 is likely involved in the maturation of immature granulocytes. Research has shown that the overexpression of KLF4 in CMPs promotes monocyte differentiation while almost completely inhibiting the generation of granulocytes, indicating that KLF4 promotes monocyte differentiation while simultaneously inhibiting granulocyte differentiation. In the bipotent promyelocytic cell line HL-60, forced overexpression of KLF4 blocks the granulocyte differentiation-promoting effect of all-trans retinoic acid (used to induce granulocyte differentiation) (32). This finding further supports KLF4’s role in inhibiting granulocyte differentiation. However, literature on the role of KLF4 in granulocytes is currently limited and further research is needed to elucidate the molecular mechanisms involved and to validate its impact on granulopoiesis *in vivo*.

## 4 The dual regulatory role of KLF4 in tumors

KLF4 exhibits a context-dependent dual role in tumor biology, functioning as a tumor suppressor in some contexts while acting as an oncogene in others. This duality is largely dependent on specific tumor types (as detailed in **Table 1**), tumor grade, and stage, as well as its role in modulating tumor microenvironment (TME) responses and therapy-induced immunity. In most solid and hematologic malignancies, KLF4 acts as a tumor suppressor by inhibiting cell proliferation and promoting apoptosis. However, in certain cancers, KLF4 demonstrates oncogenic properties, likely associated with tumor stem cell differentiation. The complex functions of KLF4 in cancer arise from its regulation across multiple molecular levels. At the transcriptional level, KLF4 expression is regulated by epigenetic mechanisms, including promoter methylation, histone modifications, and non-coding RNAs (such as microRNAs and circRNAs), which collectively determine its expression patterns in different tumors (92). For instance, in cancers such as urothelial carcinoma, renal cell carcinoma, medulloblastoma, colorectal adenocarcinoma, and gastric cancer, KLF4 is often silenced due to promoter hypermethylation, which recruits DNA-binding proteins or histone deacetylases (HDACs), leading to chromatin compaction and transcriptional repression (93–97). In contrast, in head and neck squamous cell carcinoma (HNSCC), KLF4 exhibits oncogenic functions through interactions with super-enhancers or other chromatin activation mechanisms (98). At the post-transcriptional level, KLF4 mRNA stability and translation efficiency are primarily regulated by non-coding RNAs, while RNA methylation modifications also play a crucial role, influencing RNA localization, splicing, translation, and degradation, which in turn affect cancer progression (74, 99–101). At the post-translational level, KLF4 undergoes various modifications, such as phosphorylation, acetylation, ubiquitination, and SUMOylation, which impact its stability, subcellular localization, and interactions with co-activators or inhibitors (102, 103). These regulatory mechanisms are context-specific and will be discussed further in the context of various cancers in the following sections.

**Table 1:** The role of KLF4 in major cancers, a summary of the top ten cancers by incidence (based on the latest 2024 SEER database report).

Tumor Types	Tumor Subtypes	Function of KLF4	REFERENCE
Breast Cancer	Unclassified Breast Cancer	Suppress tumors	(43–48)
	Triple-Negative Breast Cancer	Promote tumors	(43, 47, 49, 50)
	Ductal carcinoma in situ	Promote tumors	(51)
Prostate Cancer	Localized Prostate Cancer	Promote tumors	(52–54)
	Metastatic Prostate Cancer	Suppress tumors	(55)
Lung Cancer	Non-Small Cell Lung Cancer	Promote/Suppress tumors	(56–59)
	Unclassified Lung Cancer	Suppress tumors	(60, 61)
Colorectal Cancer	–	Promote/Suppress tumors	(62–69)
Melanoma	–	Promote/Suppress tumors	(70–73)
Gastric Cancer	–	Suppress tumors	(74–80)
Liver Cancer	Hepatocellular Carcinoma	Promote/Suppress tumors	(81–83)
	Perihilar Cholangiocarcinoma	Suppress tumors	(84)
Cervical Cancer	Cervical Squamous Cell Carcinoma	Suppress tumors	(85, 86)
Pancreatic Cancer	–	Promote/Suppress tumors	(87–89)
Renal Cancer	–	Suppress tumors	(90, 91)

### 4.1 Breast cancer

KLF4 role switching in breast cancer may be associated with the stage and subtype of the disease as it has been implicated in promoting the progression of low-grade primary ductal carcinoma (104) while suppressing highly aggressive triple-negative breast cancer (TNBC). As a prognostic marker in TNBC (105), KLF4 inhibits the transcription of EGFR, thereby attenuating downstream signaling pathways (e.g., MAPK and PI3K/AKT pathways). This inhibition suppresses TNBC cell proliferation, migration, and invasion, while also increasing EGFR inhibitor erlotinib sensitivity (43).

The regulation of KLF4 involves multiple upstream factors and downstream targets, affecting its transcription, splicing, and protein degradation processes. Among upstream regulators, AR and DYRK2 promote KLF4 transcription (106), while NFI-C positively regulates its expression (107). Additionally, DDX3X is responsible for splicing the primary transcript of KLF4 (108). Non-coding RNAs such as miR-7 and miR-1233-3p negatively regulate KLF4 expression (44, 109, 110). At the protein level, ATXN3 and FBXO32 modulate KLF4 stability through protein degradation pathways (111, 112).

Regarding downstream targets, KLF4 positively regulates E-cadherin, LASS2, PFKP, and S100A14 (113–116), influencing therapy response and cellular proliferation in breast cancer. These regulatory mechanisms highlight the critical role of KLF4 in determining the biological behavior of breast cancer cells.

### 4.2 Hepatocellular carcinoma

KLF4 may suppress the initiation and progression of hepatocellular carcinoma (HCC), with its expression levels closely linked to patient prognosis (117). Moreover, the expression of KLF4 in HCC tissues is generally lower than in adjacent non-tumor tissues. In HCC, KLF4 acts as a transcription factor, directly activating the expression of CD9, CD81, EpCAM, and CD133/Prominin-1 (81, 118) to suppress HCC cell proliferation through the EGFR and JNK signaling pathways. Additionally, KLF4 suppresses the growth and migration of human liver cancer cells by upregulating P-cadherin (CDH3) expression. This inhibitory effect is dependent on the regulation of the GSK-3β signaling pathway (82).

However, KLF4 also demonstrates oncogenic properties in HCC which are closely related to its cancer stem cell (CSC) maintenance capacity. MEOX2 activates KLF4 transcription, further enhancing the stemness traits of CSCs, thereby promoting liver cancer growth and recurrence (83). This contrasting regulatory effect suggests that the role of KLF4 in HCC is complex and may depend on the specific status of its upstream and downstream molecular networks.

### 4.3 Gastric cancer

KLF4 plays a crucial role in the development and progression of gastric cancer and its expression is influenced by multiple regulatory factors. In gastric cancer, KLF4 acts as a tumor suppressor. For instance, KLF4 upregulates SAMHD1, inhibiting the MAPK/p38 signaling pathway, thereby suppressing the progression of gastric cancer (75). Additionally, KLF4 exerts tumor-suppressive effects by inhibiting the transcription of CXCL8 (76). Among KLF4’s downstream target genes, iASPP, PODXL, and STK33 are negatively regulated by KLF4 (77, 119, 120), and these genes are closely associated with gastric cancer progression and prognosis.

The regulation of KLF4 is influenced by several upstream factors. CagA and TET1 inhibit KLF4 expression by methylating its promoter region (121). The transcription factor STAT5A further suppresses KLF4 transcription, promoting tumor progression, but KLF4 overexpression can counteract the oncogenic effects of STAT5A (74). Additionally, some studies suggest that KLF4 overexpression increases the sensitivity of gastric cancer cells to cisplatin and TRAIL, promoting apoptosis through DR4/DR5 upregulation, enhancing cell death induced by these treatments (78).

KLF4 is also regulated by various non-coding RNAs, since miR-32, miR-103, miR-135b-5p, and miR-155 negatively regulate KLF4 expression (79, 122–125), while LINC00673 also negatively modulates KLF4 expression (126). In contrast, the long, non-coding RNA SNHG5 positively regulates KLF4 expression and promotes gastric cancer development (122). These complex regulatory mechanisms highlight the multifaceted role of KLF4 in gastric cancer biology.

### 4.4 Colorectal cancer

Although substantial research supports the tumor-suppressive role of KLF4, its function in colorectal cancer (CRC) remains controversial. In colon cancer, KLF4 reduces autophagy in cancer cells by inhibiting RAB26 expression while promoting apoptosis and enhancing response to 5-fluorouracil (5-FU) (127). The RNA-binding protein MEX3A sustains cancer cells in an undifferentiated and proliferative state, enhancing radiotherapy resistance by suppressing KLF4 expression and activating the WNT signaling pathway (62). Additionally, KLF4 regulates chemoresistance in CRC. In oxaliplatin-resistant CRC cells, reduced KLF4 expression weakens its transcriptional repression of PiHL, resulting in PiHL upregulation and activation of the EZH2/HMGA2/PI3K/Akt signaling pathway to promote oxaliplatin resistance (63). Conversely, KLF4 expression sensitizes cancer cells to cisplatin cytotoxicity, likely through the KLF4-HMGB1/hTERT signaling axis (128).

Interestingly, KLF4 exhibits opposing roles depending on regulation by TCFL5 isoforms. TCFL5-E8 reduces KLF4 mRNA levels, potentially suppressing its tumor-promoting effects while CHA upregulates KLF4, possibly facilitating tumor progression (64). The role of miRNAs in CRC has been extensively reviewed (129), with mechanisms primarily involving the regulation of tumor proliferation, metastasis, and angiogenesis through KLF4 targeting. For example, miR-25-3p not only targets KLF4 in CRC cells but also transfers through exosomes to endothelial cells, suppressing both KLF4 and KLF2 expression (130). Conversely, miR-7-5p inhibits cancer stem cell (CSC) traits and enhances radiosensitivity in CRC by regulating KLF4 (65).

### 4.5 Non-solid tumors

**Figure 3:**
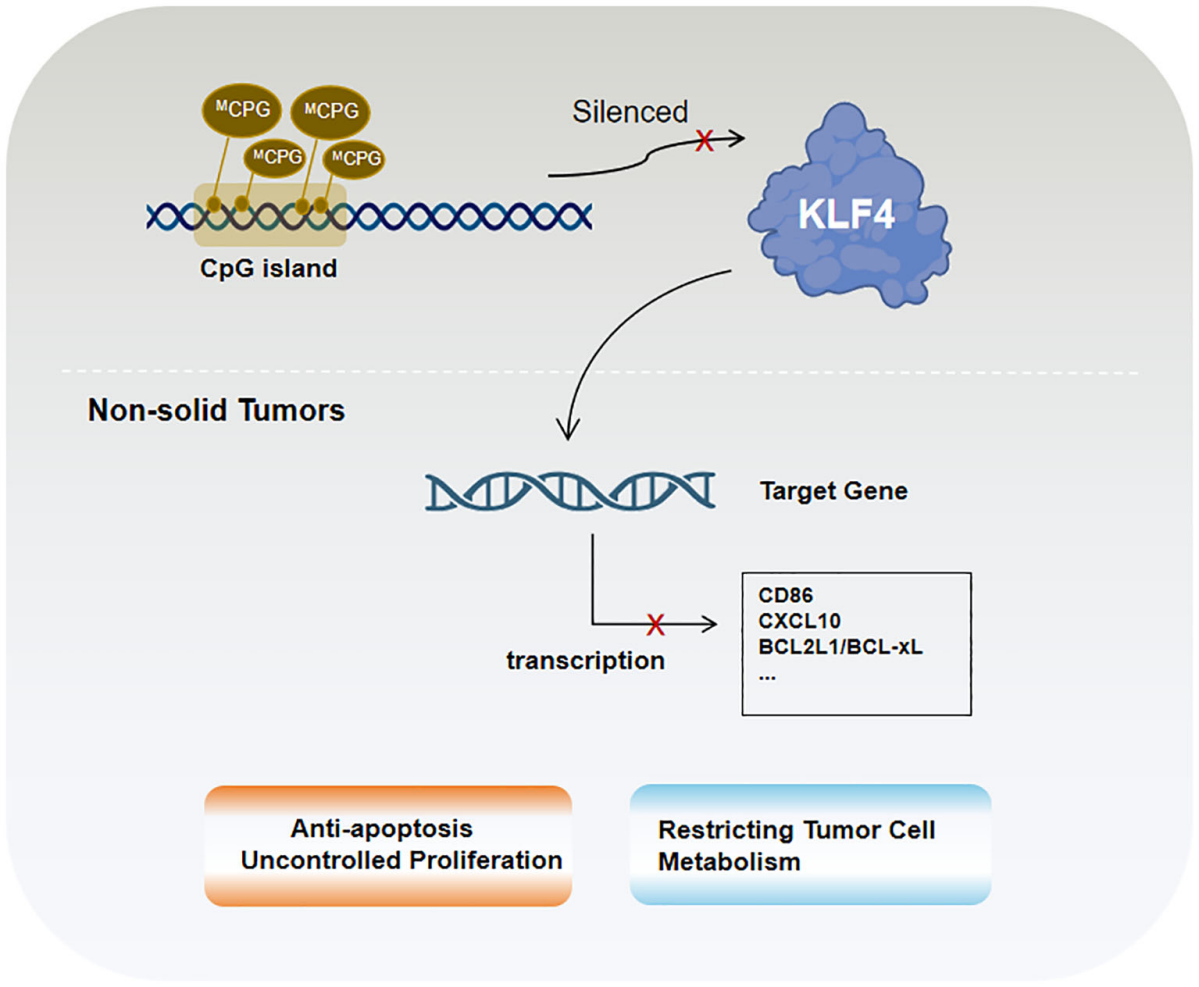
KLF4 in non-solid tumors is primarily regulated by epigenetic mechanisms, particularly CpG island methylation.

KLF4 plays an important role in non-solid tumors (e.g., lymphoma and leukemia), with its functions again dependent on tumor type and co-regulatory mechanisms. In B-cell lymphoma and various leukemia lineages, KLF4 expression is markedly reduced, potentially due to promoter hypermethylation observed in several hematologic malignancies (131, 131). This epigenetic alteration, characterized by DNA methylation, shown in **Figure 3**, is associated with KLF4 downregulation and gene silencing as observed in pediatric patients with T-cell acute lymphoblastic leukemia (T-ALL), where the loss of KLF4 significantly promotes NOTCH1-driven T-ALL development (132). In contrast, KLF4 expression is strongly upregulated in multiple myeloma cell lines (133). Besides epigenetic regulation, factors such as apoptosis, proliferation control, and microenvironmental influences significantly impact KLF4’s role in hematologic malignancies. Low KLF4 expression, on the other hand, may play a critical role in resisting apoptosis and promoting uncontrolled proliferation. One important pro-apoptotic KLF4 mechanism involves BAK1 activation, which occurs independently of caspases. In pre-B cell leukemia models, KLF4 enhances apoptosis by suppressing anti-apoptotic proteins BCL2L1/BCL-xL (134). Additionally, in cHL, KLF4 is repressed by Notch1 and lacks activation by PU.1, further amplifying its suppression (31, 135, 136).

In B-cell acute lymphoblastic leukemia (B-ALL) mouse models, KLF4 inhibits ABL-induced pre-B cell transformation by inducing apoptosis and CDKN1A-mediated cell cycle arrest. Studies have shown that co-treatment with the tyrosine kinase inhibitor imatinib and KLF4 overexpression enhances apoptotic effects, suggesting their synergistic role in promoting tumor cell death. KLF4 also negatively regulates CD86 and CXCL10 (137), thereby reducing the recruitment of bystander immune cells, such as T lymphocytes, to tumor cells. Furthermore, KLF4 interacts with other critical factors in hematologic malignancies. In Burkitt lymphoma (BL), KLF4 acts as a tumor suppressor by reducing MYC expression and its downstream target EZH2 while activating tumor suppressors such as TXNIP (137). These effects collectively decrease reactive oxygen species (ROS)-induced damage and constrain tumor cell metabolism. In most cases of acute myeloid leukemia (AML), the homeobox transcription factor CDX2 promotes leukemogenesis by suppressing KLF4 (137). Moreover, in follicular lymphoma (FL), diffuse large B-cell lymphoma (DLBCL), and BL, low KLF4 expression is likely a prerequisite for high MSC/ABF-1 expression. MSC/ABF-1 inhibits E47/E12 transcriptional activity, silencing B-cell-specific gene expression programs and further enhancing tumor cell proliferation (133).

## 5 The multifaceted role of KLF4 in the tumor immune microenvironment

The interplay between tumors and the immune system is highly complex. On one hand, the immune system plays an anti-tumor role by recognizing and eliminating abnormal cells, including cancer cells. On the other hand, tumors can evade immune surveillance and attack by utilizing immune escape mechanisms and modulating immune checkpoints. The role of KLF4 in anti-tumor immunity is crucial and deserves special attention as studies have shown that, in hepatocellular carcinoma (HCC), KLF4 is significantly associated with CD8+ T cells, Th1 cells, dendritic cells (DCs), B cells, natural killer (NK) cells, macrophages, and their molecular markers (138). KLF4 plays an important role in regulating the differentiation and function of CD8+ T cells, influencing their ability to recognize and kill tumor cells. Additionally, KLF4 is closely related to macrophage polarization, regulating their polarization toward the pro-inflammatory M1 type or the anti-inflammatory M2 type and thereby affecting tumor immune escape and progression. KLF4 is also involved in regulating inflammatory responses, determining the immune status of the TME by balancing pro-inflammatory and anti-inflammatory factors. These actions reveal the complex role of KLF4 in tumor immunity, warranting further investigation.

### 5.1 KLF4 promotes CD8+ T cell differentiation in tumors

CD8+ T cells play a critical role in tumor immunity. As cytotoxic T lymphocytes, they can directly recognize and kill tumor cells, thus exerting anti-tumor effects. CD8+ T cells play an essential defense role in immune surveillance of tumors (139) and in many types of cancer, CD8+ T cell infiltration correlates with improved patient survival. Given that KLF4 has been shown to significantly influence CD8+ T cell proliferation and differentiation in normal T cell biology, it raises the question of whether KLF4 similarly affects CD8+ T cell differentiation in the tumor microenvironment (TME), thereby impacting anti-tumor efficacy. Studies have indicated that in HCC patients with high KLF4 expression, the tumor immune microenvironment (TIME) is characterized by increased CD8+ T cell infiltration, suggesting a potential correlation between KLF4 expression and CD8+ T cell infiltration (138).

Immune-suppressive factors present in the TME, such as TGF-β, IL-10, and tumor-associated macrophages (TAMs), indirectly affect the anti-tumor capacity of CD8+ T cells. In prostate cancer, during T cell receptor activation, KLF4-deficient TAMs inhibit the expression of ELF4 and KLF4, releasing initial CD8+ T cells from quiescence and activating pro-inflammatory and pro-atherosclerotic pathways, leading to CD8+ T cell proliferation and enhanced immune response that suppresses prostate cancer growth (140). The removal of CD8+ T cells eliminates the inhibitory effects of KLF4 deficiency on prostate cancer growth. The specific molecular mechanism may involve mTOR, which inhibits KLF4 expression via downstream ERK and PI3K signaling. Additionally, research has shown that KLF4 suppresses the recruitment of CD8+ T cells to the TME via the Hedgehog signaling pathway, further contributing to immune suppression in the TME, likely by inhibiting the production of C-X-C Motif Chemokine Ligand 9 (CXCL9) and CXCL10 in TAMs (141).

CD8+ T cell exhaustion is a phenomenon observed during chronic inflammatory responses in persistent viral infections or cancer (142–144). Exhausted CD8+ T cells, due to prolonged exposure to antigens and inflammatory stimuli, gradually lose proliferation, cytokine secretion, and cytotoxic killing capacities. Exhaustion is regulated by the upregulation of several inhibitory receptors, such as PD-1, LAG-3, and TIM-3, which suppress T cell function (145–147). Tumor cells contribute to the inhibition and exhaustion of CD8+ T cells by upregulating immune checkpoint molecules, antigen loss, and altering the tumor microenvironment (148). KLF4 is closely associated with genes regulating immune checkpoints, particularly those involved in T cell exhaustion, as elevated levels of immune checkpoint molecules in the TME may increase KLF4 expression, resulting in diminished T cell function and promoting exhaustion. Additionally, some studies have indicated that KLF4 can activate and reactivate exhausted CD8+ T cells, promoting their differentiation into transient effector subpopulations and enhancing their anti-tumor activity (149). In melanoma, immunotherapy targeting the PD-1 receptor can upregulate KLF4 expression, thereby improving the survival rate of melanoma patients. The underlying mechanism may involve an increase in KLF4 expression on CD8 tumor-infiltrating lymphocytes (TILs) and an elevated number of transient effector CD8+ T cells expressing KLF4. In tumor-specific CD8+ T cells, high KLF4 expression upregulates AP-1 family factors, such as c-Jun, conferring transient effector characteristics upon CD8+ T cells (150–152). Research has demonstrated that c-Jun and BATF (basic leucine zipper ATF-like transcription factor), enhance CAR T cell effector function and even confers resistance to T cell exhaustion (152). This is consistent with KLF4’s role in upregulating c-Jun, underscoring KLF4 as critical for maintaining CD8+ effector function. In tumor-specific CD8+ T cells, KLF4 enhances effector function by regulating the expression of AP-1 family factors, such as c-Jun, thereby counteracting terminal exhaustion. The absence of KLF4 impairs CD8+ T cell differentiation and function, making them more susceptible to exhaustion and weakening the anti-tumor response.

### 5.2 KLF4 promotes macrophage polarization in tumors

In addition to CD8+ T cells, macrophage content is significantly higher in the tumor tissues of HCC patients with high KLF4 expression compared to low KLF4 expression (138). Tumor-associated macrophages (TAMs) can be classified into M1 and M2 types: M1 macrophages exhibit pro-inflammatory characteristics, activating immune responses and killing tumor cells, while M2 macrophages tend to be anti-inflammatory, promoting tumor growth and metastasis. Thus, macrophages play a dual role in tumor immunity, with their function depending on their polarization state (153, 154). KLF4 influences macrophage polarization toward either the M1 or M2 phenotype, acting as a key regulatory factor that not only induces M2 genetic programming but also maintains the balance by inhibiting M1 target gene expression. Additionally, KLF4 plays an essential role in maintaining the immune rhythm of macrophages; changes in its expression levels can therefore negatively affect immune function, leading to tumorigenesis (155).

Research has demonstrated KLF4 regulation of M2 polarization through its SUMOylation status, with specific protease SUMO-specific Protease 1 (SENP1) responsible for de-SUMOylating KLF4. This process drives macrophage polarization toward the M1 type, with the SENP1-KLF4 axis playing a key role in conjunction with the NF-κB signaling pathway. Moreover, KLF4 cooperates with Stat6 to induce M2 genetic programming and inhibits M1 target gene expression by sequestering co-activators necessary for NF-κB activation (156). In the Hedgehog signaling pathway, tumor cells secrete sonic hedgehog (SHH), a Hedgehog ligand that drives TAMs toward M2 polarization (156).

In diverse tumor types, KLF4 expression is closely associated with macrophage polarization. In liver cancer, miR-206 targets KLF4, suppressing its expression and promoting M1 macrophage polarization, thereby effectively preventing hepatocellular carcinoma. This mechanism may involve the KLF4/C-C Motif Chemokine Ligand 2 (CCL2)/C-C Motif Chemokine Receptor 2(CCR2) axis (157). In gastric cancer, exosomes derived from gastric cancer cells carry the long non-coding RNA (lncRNA) HCG18, which reduces the levels of miR-875-3p, thereby increasing KLF4 expression and promoting M2 macrophage polarization (158). In non-small cell lung cancer (NSCLC), KLF4 expression is upregulated and closely associated with macrophage infiltration and M2 polarization, while miR-34a-5p targets KLF4 and reverses macrophage polarization, regulating immune molecules such as IL-1β, thus showing therapeutic potential for NSCLC (58).

**Figure 4:**
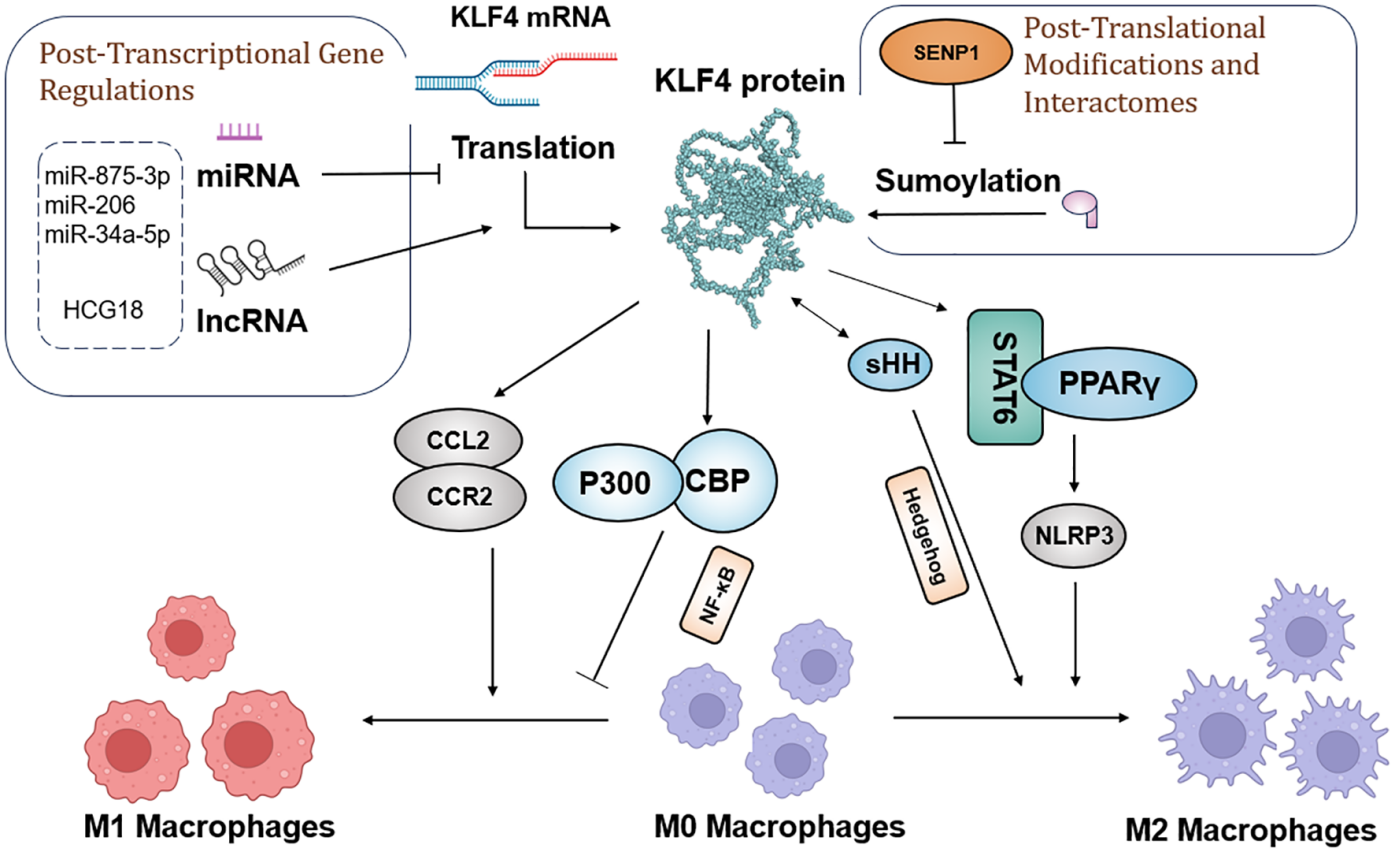
Schematic model of the molecular mechanisms by which KLF4 regulates macrophage polarization in the tumor environment, before and after transcription and translation.

Furthermore, KLF4 plays an important role in inhibiting pro-inflammatory genes in M2 macrophages. Its absence weakens IL-4’s ability and suppresses M1 target genes. In immune-related thrombocytopenia (ITP), low-dose DAC promotes M2 macrophage polarization and inhibits inflammatory responses by enhancing KLF4’s binding to the PPARγ promoter (159). Overall, since KLF4 plays a crucial role in regulating macrophage polarization and function, understanding its mechanisms will contribute to the development of new tumor therapeutic strategies. The molecular mechanism by which KLF4 regulates macrophage polarization is shown in **Figure 4**.

### 5.3 KLF4 and the inflammatory response

Macrophage activation plays a central role in the onset and progression of chronic inflammation. Pro-inflammatory factors such as interferon-γ (IFN-γ), lipopolysaccharide (LPS), and tumor necrosis factor-α (TNF-α) promote macrophage activation, while anti-inflammatory factors like TGF-β suppress inflammation. This regulatory mechanism is crucial in both inflammatory diseases and cancer progression, with KLF4 playing a significant role in regulating macrophage pro-inflammatory responses, potentially acting as a key target in the TGF-β1/Smad3 signaling pathway (160). Studies have shown that KLF4 is essential for the expression of the macrophage activation marker iNOS, induced by IFN-γ or LPS, a process partially mediated by KLF4’s interaction with p65 (RelA), a member of the NF-κB family (30). Additionally, KLF4 is an IFN-γ target gene in cancer cell lines, with its function mediated by Stat1. Further research by Tetreault et al. demonstrated that KLF4 overexpression in mouse esophageal epithelial cells activates pro-inflammatory factors such as TNF-α, CXCL5, Granulocyte Colony-Stimulating Factor(G-CSF), and IL-1α, a process that also relies on NF-κB, ultimately leading to the development of esophageal squamous cell carcinoma (161). Hp infection can induce the expression of miR-135-5b by activating the pro-inflammatory NF-κB signaling pathway. miR-135-5b binds to the 3’ UTR of the Klf4 gene, leading to reduced Klf4 expression and promoting the development of gastric cancer (124).

## 6 KLF4 and tumor-targeted therapies

Studies have shown that KLF4 activity can be modulated by exogenous drugs, indicating novel therapeutic approaches for cancer treatment. Several studies have reported that known compounds exert antitumor effects by acting on KLF4 in diverse types of cancer. For instance, in triple-negative breast cancer, the small molecule inhibitor WX2-43 blocks KLF4 methylation by inhibiting the Protein Arginine Methyltransferase 5 (PRMT5) -KLF4 interaction, showing significant antitumor efficacy (162). Kenpaullone inhibits KLF4 by suppressing CDK1/cyclin B and GSK-3β, reducing proliferation and migration of breast cancer cells in canine mammary cancer and inducing cancer cell death, although its effects in humans require further investigation (163). In colorectal cancer, sulforaphane induces KLF4 and enhances KLF4-p21 signaling, contributing to the inhibition of cancer cell growth and promoting differentiation (164). Regorafenib, a multi-kinase inhibitor, significantly suppresses tumor growth and angiogenesis in hepatocellular carcinoma (HCC), with effects similar to those of DC-101 (an anti-VEGFR2 antibody). However, Regorafenib is more effective in activating T cells and promoting M1 macrophage polarization. It also reverses M2 macrophage polarization by inhibiting p38 kinase phosphorylation and downstream Creb1/KLF4 activity (165). The small molecule compound APTO-253, which induces KLF4, was confirmed to have antitumor activity in a 2015 Phase I multicenter study (166). Recent research has also demonstrated that APTO-253 enhances immune responses in leukemia, rendering cancer cells more susceptible to NK cell-mediated termination. Increased KLF4 activity leads to elevated MICA expression on AML cells which, in turn, strengthens NK cell-mediated immune surveillance (167). KLF4 is also a downstream protein of Peroxisome Proliferator-Activated Receptor Gamma (PPARγ) and its expression can enhance PPARγ levels. Pioglitazone, a PPARγ agonist, stabilizes KLF4 protein by activating the AKT pathway and reducing KLF4 ubiquitination, suggesting its potential as a KLF4-targeting drug (168, 169). Decitabine can enhance KLF4’s binding to the regulatory region of PPARγ, potentially increasing PPARγ transcriptional activity. Additionally, as a KLF4 methylation inhibitor, decitabine upregulates KLF4 expression by inhibiting methylation of the KLF4 promoter, possibly through suppression of the EMT pathway (170). Moreover, KLF4 can sensitize cancer cells to several established therapies, including cisplatin (124, 128, 171–173), cetuximab (22), mesalazine (174), and gefitinib (175), suggesting the potential for combinatory use with other anticancer drugs in future clinical trials. In summary, drugs targeting KLF4 are gradually being discovered for their potential to impact malignant tumors, but further mechanistic studies and clinical validation are necessary. Drugs targeting KLF4 and their mechanisms are listed in **Table 2**.

**Table 2:** Drugs targeting KLF4 and their mechanisms.

	Compounds	Mechanism	Reference
KLF4	WX2-43	Promotes PRMT5-KLF4 interaction	(162)
	Kenpaullone	Suppress CDK1/cyclin B and GSK-3β	(163)
	Sulforaphane	Enhances KLF4-p21 signaling	(163)
	Regorafenib	Regulates Creb1/KLF4 Promotes M1 macrophage polarization	(165)
	APTO-253	Makes cells susceptible to NK cells	(167)
	Pioglitazone	Activates the AKT pathway and reduces KLF4 ubiquitination	(168, 169)
	H89 and 14–22 Amide (PKI)	Enhance KLF4 transcription	(176, 177)
	Decitabine	Suppress EMT pathway	(170)

## 7 Conclusion and perspectives

Previous reports indicate that KLF4 and its interacting molecules and pathways are involved in various types of cancer, leading to a reasonable hypothesis that KLF4 can serve as a potential target for cancer therapy. However, most experimental results have been based on simple assays performed at either the RNA or protein level. While KLF4 may exhibit high expression at the transcriptional level, its protein expression may not correspond due to various regulatory mechanisms, leading to the mistaken assumption that it has an inhibitory (or promotive) effect. Additionally, the lack of a ligand-binding domain renders the development of targeted drugs particularly challenging. Nevertheless, using KLF4 as a biomarker for tumor diagnosis and prognosis presents a promising option. However, given that KLF4 displays divergent, and at times even completely opposite, expression patterns in different stages and environments of tumors, its specific role in clinical and pathological grading and staging of cancer requires further exploration.

This article focuses on KLF4’s functions in various immune cells and its relationship with cancer and immunity. KLF4 exerts profound effects on multiple immune cells under physiological conditions and, in tumors, it primarily influences progression by regulating T cell subset differentiation and macrophage polarization states to regulate therapy resistance. In recent years, immunotherapy has made remarkable strides and has become a breakthrough in cancer treatment by activating or modulating the immune response to recognize and attack tumor cells. Notably, Adoptive Cell Transfer (ACT) and ICI therapies have made significant progress in both solid and hematologic malignancies by either directly modifying and expanding autologous T cells or relieving tumor-induced T cell suppression, thereby actively or passively modulating the immune response. This underscores the potential of leveraging immune cells, particularly T cells, to eliminate tumor cells. Moreover, the immunological roles of KLF4 in cancer help clarify the interaction between innate and adaptive immunity within the tumor microenvironment, providing new opportunities for developing strategies to further enhance antitumor immunity.
